# The Birds of the Wood

**Published:** 1859-11

**Authors:** 


### THE BIRDS OF THE WOOD.

The true uses of the beautiful are to happify man, hence we shall never fail to find throughout the wide empire of the benificent Father of us all, that beauty has its uses; or if those uses are not known to us now, a closer observation will discover them. Then spare, oh spare, the beautiful birds of the early spring time, and of the maturer summer; for while they delight us with their sweet, glad twitterings, they perform a toil all day, which sturdy man, with all his wisdom and all his power would be wholly inadequate to accomplish. Time out of mind have we been told that the birds were the worst enemies of the hard working tiller of the soil, and with that impression, millions of these loving warblers have been remorsely, yes, gladly destroyed. But not long ago, a farmer, as observant as he was humane, shot a yellow-bird in his field, in order to convince a neighbor that birds were actually useful rather than destructive. On examining its little stomach, they found it contained two hundred wevils and only four grains of wheat. Birds like our domestic fowls, thrive on flesh, and are the voracious destroyers of insects.

But as sweetness of character is the steady attendant of benevolence in men, so there is a kindness in the little bosom of the feathered songster, which well accords with its bonny plumage, its beautiful voice, and its sterner utilities.

The correspondent of a Washington paper relates, that noticing an extraordinary commotion near a bird's nest, he found that a mother-bird had been caught by the wing among the twigs of a tree; her cries brought others, and when her efforts for release were unavailing, the other birds flew away, but after awhile returned, each bearing an insect of some kind, or other article of food, in its bill; some gave to the mother, others gave to her half-grown nestlings near by. When the gentleman released the mother, there seemed to be a universal jubilation for a short time, when the others flew away, and the mother-bird nestled among her young ones.

Who that reads this beautiful incident will ever hurt a bird again, or allow children, or any person under them, to do it?

And if the little birds thus help one another in trouble, let not man, with his high relationship to angels, ever fail in aiding an unfortunate brother in his sorrow, in his poverty, or in the hour of crushing trial, or wasting illness.

---

### HOW TO COUNT.

"Mr. Smith, why have you kept me waiting to let you in, until three o'clock in the morning?"

"It's a mistake, my dear, you don't know how to count, for I heard the clock strike one just now, two or three times."

